# Association of depressive symptoms with sweat chloride variability and medication possession ratio during long-term Elexacaftor/Tezacaftor/Ivacaftor therapy in adolescents and young adults with cystic fibrosis

**DOI:** 10.3389/fped.2026.1888421

**Published:** 2026-08-31

**Authors:** Michael Lorenz, Mariya Pavlova, Katja Erler, Anika Nader, Anne Moeser

**Affiliations:** Cystic Fibrosis Centre, Department of Paediatrics, Jena University Hospital, Friedrich-Schiller-University, Jena, Germany

**Keywords:** adherence, cystic fibrosis, depression, psychological screening, sweat chloride

## Abstract

**Introduction:**

Elexacaftor/tezacaftor/ivacaftor (ETI) sustainably improves lung function (FEV₁), quality of life and sweat chloride (SC) levels in people with cystic fibrosis (pwCF). Psychological comorbidities may impair treatment adherence. This study assessed ETI adherence using SC and medication possession ratio (MPR) and evaluated the impact of depressive symptoms.

**Methods:**

In this retrospective observational study, 34 pwCF aged 12–21 years (median 17.3) initiating ETI between 2020 and 2022 were included. SC and FEV₁ were measured at baseline and annually over four years. Depressive symptoms were screened annually using the PHQ-9 (score ≥7). Adherence was assessed by MPR (1.0 = full medication supply). Patients with prior CFTR modulator therapy, insulin-dependent diabetes or organ transplantation were excluded.

**Results:**

Fifteen pwCF (44.1%) screened positive for depressive symptoms (mean PHQ-9 scores 11 vs. 2). Median SC decreased significantly from 102.5 mmol/L (98–106.5) at baseline to 46.5 mmol/L (32.5–57.8) in year 1 (*p* < 0.001) and remained stable thereafter. PwCF with depressive symptoms demonstrated greater SC variability during follow-up, with a trend toward higher SC levels at follow-up visit 4 (between-group *p* = 0.056), while the within-group increase from FV1 to FV4 was significant (*p* = 0.012)., MPR declined significantly from 0.95 to 0.75 (*p* < 0.05). FEV_1_ improved significantly in year 1 compared to baseline (+11%, *p* < 0.001) and remained stable in both groups.

**Conclusion:**

ETI leads to sustained improvements in lung function and sweat chloride in pwCF. A substantial proportion of patients screened positive for depressive symptoms, which was associated with increased long-term variability in sweat chloride and declining MPR but not lung function. Structured psychological monitoring and targeted interventions may be essential to support adherence and long-term treatment success.

## Highlights

- Depressive symptoms drive long-term ETI adherence decline in adolescents with CF.
- Sweat chloride variability may signals suboptimal modulator adherence.
- Stable lung function may mask early treatment disengagement.

## 1 Introduction

Cystic fibrosis (CF) requires sustained engagement with a complex, time-intensive daily regimen (airway clearance, inhaled and oral medications, nutrition, exercise) Suboptimal adherence remains a persistent barrier to achieving the full benefit of therapy. While psychological and behavioural adherence-support interventions have been developed—particularly for inhaled therapies—the evidence is still limited and heterogeneous, underscoring the need to better define modifiable drivers of adherence and to evaluate scalable support strategies in routine care settings. (1) In parallel, the rapid expansion of CFTR modulator use has shifted adherence research priorities: a recent systematic review highlights both the relative paucity of real-world adherence data for CFTR modulators and the variability in adherence definitions and measurement approaches [most commonly pharmacy refill metrics such as medication possession ratio (MPR) or proportion of days covered]. (2) Objective adherence metrics are clinically meaningful in CF. For example, pharmacy-derived composite MPR has been linked to subsequent health-care utilization and cost, supporting its role as a pragmatic adherence indicator for longitudinal observational studies (3). Complementary work using objectively measured adherence data has also emphasized the multi-determinant nature of adherence behaviour (capability, opportunity, motivation) and the value of theory-informed approaches to intervention development. (4) Across the broader CF home-management program, adherence estimates vary substantially by treatment component and by the assessment method used (objective vs. self-report), reinforcing the importance of triangulating adherence measures wherever feasible. (5)

The introduction of highly effective CFTR modulator therapy—most notably the triple combination elexacaftor/tezacaftor/ivacaftor (ETI)—has fundamentally changed CF care. Clinical trial evidence demonstrates that ETI can produce fast improvements in clinical and patient-reported outcomes, including lung function, respiratory quality of life, and sweat chloride as a biomarker of CFTR function. (6, 7) However, as with other chronic therapies, the long-term benefit in real-world practice depends on consistent medication access and use. This is particularly relevant for adolescents and young adults, a developmental period associated with shifting responsibility for self-management, changing routines, and increased vulnerability to competing social and educational demands—factors that can destabilize adherence patterns over time (3, 5).

Psychological comorbidities are increasingly recognized as central to CF outcomes and self-management. International consensus statements from the Cystic Fibrosis Foundation and the European Cystic Fibrosis Society recommend routine screening and structured care pathways for depression and anxiety in CF, reflecting the high prevalence and clinical relevance of depressions in this population (8). Beyond prevalence, multiple studies and reviews indicate that depressive symptoms and broader psychological diseases are associated with poorer adherence behaviours across CF treatment modalities (9–13). Evidence in young people and families suggests that both individual and family-level psychological factors (e.g., depressive symptoms, treatment beliefs, relational context) may influence adherence, highlighting a need for integrated models that capture behavioural, psychosocial, and contextual determinants rather than relying on a single explanatory factor. (11, 13) In this context, adherence should be conceptualized not only as a medication-taking behaviour but as an outcome shaped by psychological wellbeing, perceived treatment burden, beliefs about necessity and concerns, and the practical realities of daily life with CF (4, 9, 13).

Given the transformative clinical potential of ETI and the plausible vulnerability of adherence to depressions, there is a strong rationale to evaluate adherence longitudinally using organ-specific outcome measures. Pharmacy refill-based MPR provides a pragmatic, reproducible estimate of medication availability, while sweat chloride offers a biologically grounded marker of CFTR modulator effect that may be informative when interpreted alongside refill data (2, 3, 6). Building on this framework, the present study assessed ETI adherence in adolescents and young adults initiating ETI by combining MPR with sweat chloride measurements and examined the impact of depressive symptoms assessed through standardized screening (PHQ-9) at annual follow-up visits over several years. The overarching objective was to clarify how depressive symptoms may relate to adherence trajectories in the ETI era and to inform structured monitoring and targeted support strategies to optimize long-term treatment success (8).

## 2 Methods

### 2.1 Study design and population

We conducted a retrospective observational cohort study at the Cystic Fibrosis Centre, Department of Paediatrics, Jena University Hospital (Friedrich Schiller University, Jena, Germany). We included people with cystic fibrosis (pwCF) aged 12–21 years who initiated elexacaftor/tezacaftor/ivacaftor (ETI) between 2020 and 2022 (Table 1). Exclusion criteria were: (i) prior CFTR modulator therapy with ivacaftor, lumacaftor, or tezacaftor; (ii) insulin-dependent diabetes; and (iii) history of organ transplantation.

**Table 1:** Definition of abbreviations: y, years; CFTR, cystic fibrosis transmembrane conductance regulator; ETI, elexacaftor/tezacaftor/ivacaftor; FEV1pp, forced expiratory volume in one second; IQR, interquartile range.

Variable	Without depressive symptoms *n* = 19 (%)	With depressive symptoms *n* = 15 (%)	*p*-value
Gender female	13 (68.4%);	10 (66.7%);	1.000
male	6 (31.6%)	5 (33.3%)	
Age (y)	16.2 (15.05–18.85)	18.2 (16.6–20.10)	0.259
CFTR Genotype			
F508/F508 (F/F)	F/F: 9 (47.4%)	F/F: 9 (60.0%);	0.510
F508/Minimal function (F/MF)	F/MF: 10 (52.6%);	F/MF: 6 (40.0%)	
Chron. Pseudomonas aeruginosa	4 (21.1%)	2 (13.3%)	0.672
	Median ± IQR	Median ± IQR	
Treatment duration ETI (months)	42.00 (37.00–46.50)	44.00 (39.00–45.00)	0.414
FEV1 initial (%)	95.00 (89.00–102.00)	91.00 (79.00–101.00)	0.677
DS = PHQ-9 score	2.00 (1.00–4.00)	11.00 (8.00–13.50)	<0.001*
Medication possession ratio First Year	1.00 (1.00–1.00)	1.00 (0.92–1.00)	0.296
Pulmonary exacerbations rate per year (two years prior)	2.50 (2.00–3.00)	2.50 (2.00–2.75)	0.831

Data presented as median with IQR for continuous variables and *n* (%) for categorical variables;.

DS, depressive symptoms (PHQ-9 score ≥7);

Statistical tests: Fisher's exact test for categorical variables; Mann–Whitney U test for continuous variables.

***p < 0.001.

### 2.2 The clinical measurements

Sweat chloride concentration (SC) was obtained before ETI initiation (initial) and then annually at routine follow-up visits (Visit 1–4. Lung function was assessed in parallel at the same time points using forced expiratory volume in one second (FEV₁. depressive symptoms were screened annually using the Patient Health Questionnaire-9 (PHQ-9).for group allocation, the maximum PHQ-9 value observed over the follow-up period was used. The ≥7 threshold was specified *a priori* as a sensitive screening threshold, consistent with the German COACH-MI programme, in which a PHQ-9 or GAD-7 score ≥7 triggers a recommendation for psychological support in adolescents with chronic conditions (14–16). Because it was externally pre-specified rather than optimised within our sample, it is not subject to the small-sample bias of data-driven cut-off selection (17); the conventional ≥10 threshold was additionally analysed as a sensitivity analysis (Supplementary Table S1) (18). The GAD-7 was not consistently available in this retrospective dataset, so anxiety could not be assessed. In a subset of 15 pwCF, we additionally recorded the presence of predefined familial burden factors, including: (i) single-parent household/separated parents, (ii) socioeconomic strain, and (iii) parental mental health problems.

The adherence to ETI has been assessed with a pharmacy refill–based medication possession ratio (MPR) A MPR of 1.0 represents full medication supply coverage over the respective interval. Medication packages are dispensed on a quarterly basis (i.e., providing a 3-month supply), reflecting a prescribing practice that may represent a specific characteristic of the German healthcare system In addition, SC was used as a complementary objective biomarker reflecting pharmacodynamic CFTR response to ETI over time.

Pulmonary exacerbations (PE) were defined as the need for additional oral or intravenous antibiotic treatment as indicated by at least two of the following: change in sputum volume or colour; increased cough; increased malaise, fatigue or lethargy; anorexia or weight loss; ≥10% decrease in pulmonary function; radiographic changes; and/or increased dyspnoea. We analyzed the two years prior to the launch of ETI (Visit Number -2 and -1 (19);

### 2.3 Statistical analysis

All statistical analyses were performed using SPSS version 30 (IBM Corp., Armonk, NY, USA). Baseline characteristics between groups were compared using Fisher's exact test for categorical variables and Mann–Whitney U test for continuous variables, as data were not normally distributed. Longitudinal changes in clinical parameters (sweat chloride, FEV1, medication possession ratio, and pulmonary exacerbations) were analyzed using Friedman test to assess overall change over time, followed by Wilcoxon signed-rank tests for pairwise comparisons between time points. Between-group differences at each visit were compared with the Mann–Whitney U test. In *post hoc* analyses, all comparisons were repeated after reclassifying participants at the conventional PHQ-9 ≥ 10 threshold, and within-subgroup changes from FV1 to FV4 were tested with Wilcoxon signed-rank tests; exact tests are reported for small groups. Owing to the small, non-normally distributed sample, no formal interaction model was fitted, and analyses beyond the primary within-group comparisons are considered exploratory. Continuous variables are presented as median with interquartile range [IQR], and categorical variables as number and percentage. A two-tailed *p*-value <0.05 was considered statistically significant, with trends indicated at *p* < 0.10.

## 3 Results

### 3.1 Entire cohort

**Figure 1:**
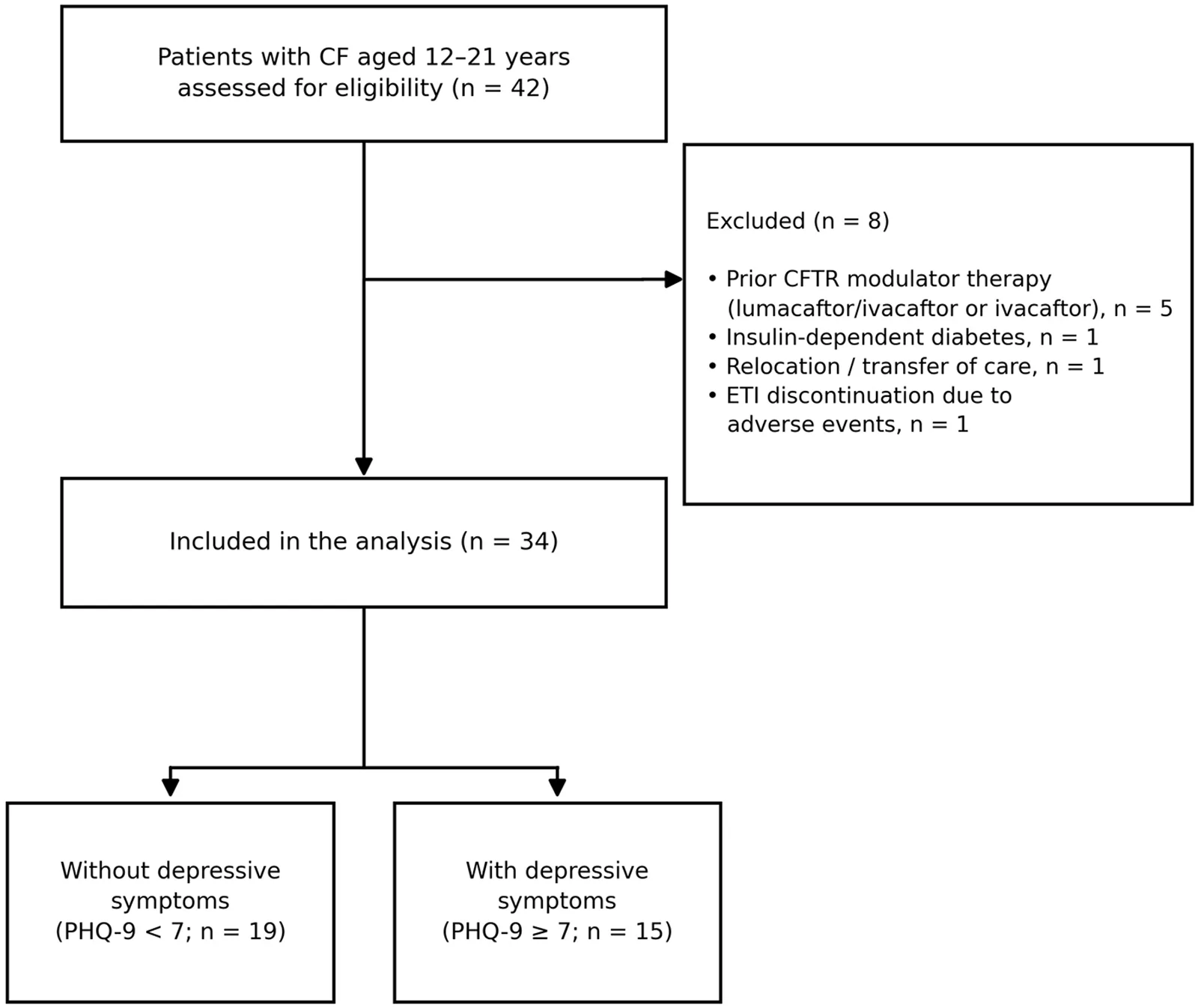
Participant flowdiagram.

Fourty-two adolescents and young adults with CF were assessed for eligibility, 8 were excluded — 5 for prior CFTR modulator therapy (lumacaftor/ivacaftor or ivacaftor), 1 for insulin-dependent diabetes, 1 for relocation or transfer of care, and 1 for discontinuation of ETI due to adverse events — leaving 34 participants for analysis (Figure 1). Based on the maximum PHQ-9 score over follow-up, 19 participants (55.9%) were classified as without depressive symptoms (PHQ-9 < 7) and 15 (44.1%) as with depressive symptoms (PHQ-9 ≥ 7). Baseline demographic and clinical characteristics were comparable between the two groups (Table 1).

### 3.2 Sweat chloride

**Figure 2:**
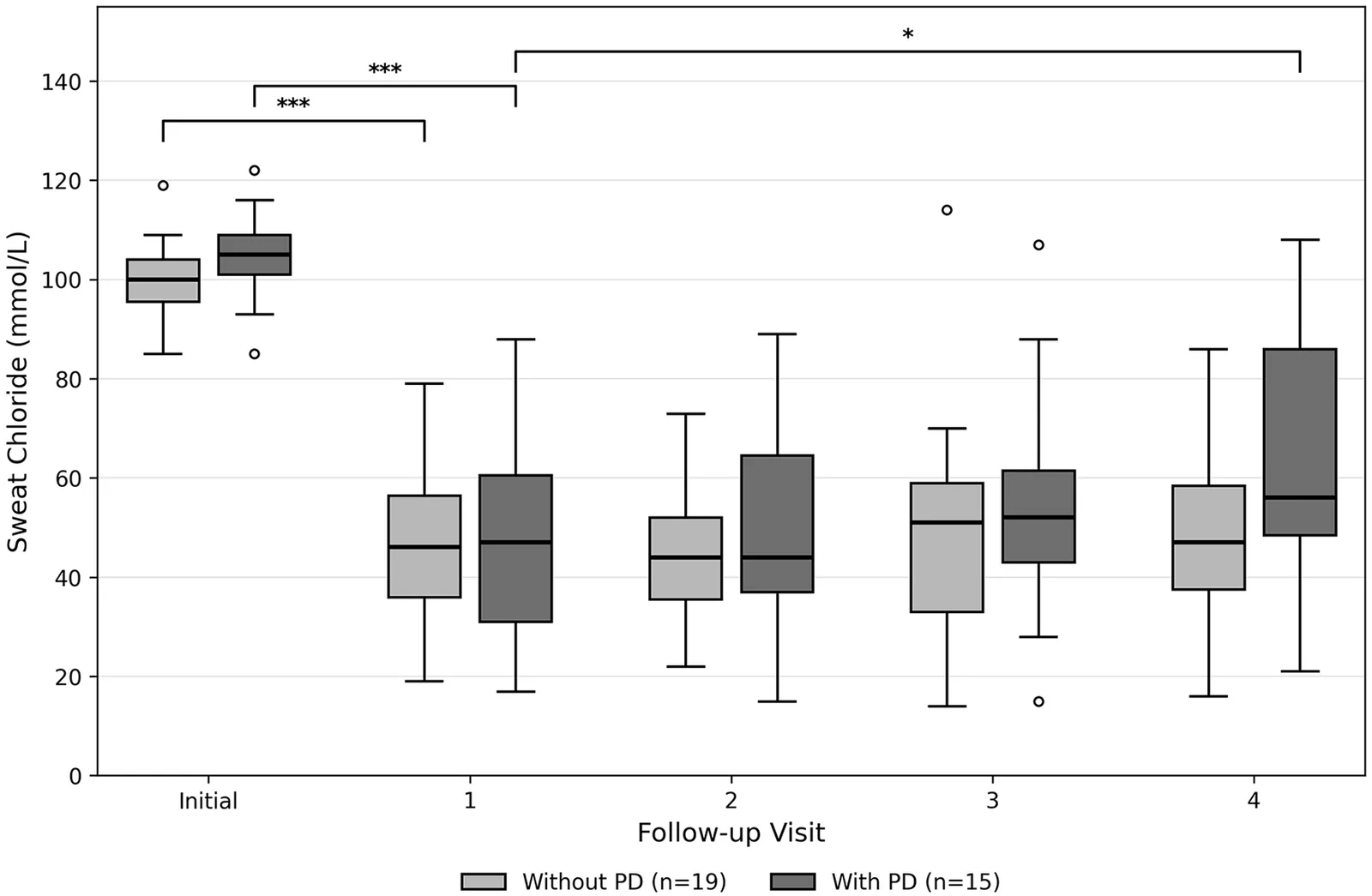
Sweat chloride measurements at baseline and during follow-up visits (FV1-4) after the start of ETI DS, depressive symptoms (PHQ-9 score ≥7); *p < 0.05; ***p < 0.001.

Sweat chloride concentrations showed significant reductions following modulator therapy initiation in both patient groups (Figure 2). In patients without depressive symptoms (*n* = 19), median sweat chloride decreased from 100 mmol/L (IQR 96–104) at baseline to 46 mmol/L (IQR 36–56) at follow-up visit 1 (FV1)1 (*p* < 0.001). Similarly, patients with depressive symptoms (*n* = 15) demonstrated a reduction from 105 mmol/L (IQR 101–109) to 47 mmol/L (IQR 31–60) at FV1 (*p* < 0.001). Sweat chloride levels remained stable throughout subsequent follow-up visits (FV1–4) in patients without depressive symptoms (*p* = 0.323–0.573 vs. FV1). However, patients with depressive symptoms showed a significant increase at FV4 (56 mmol/L, IQR 48–86; *p* = 0.012 vs. FV1). In between-group comparisons (Mann–Whitney U), SC did not differ significantly at any visit; the FV4 difference was a non-significant trend (47.0 vs. 56.0 mmol/L; *p* = 0.056) (Table S2). The higher and more variable SC at FV4 in the depressive-symptoms subgroup is interpreted as a possible marker of reduced adherence; CFTR-function, pharmacokinetic and dietary factors may also contribute (Supplementary Table S2). The overall time effect was highly significant in both groups (Friedman test: *p* < 0.001).

### 3.3 Lung function

**Figure 3:**
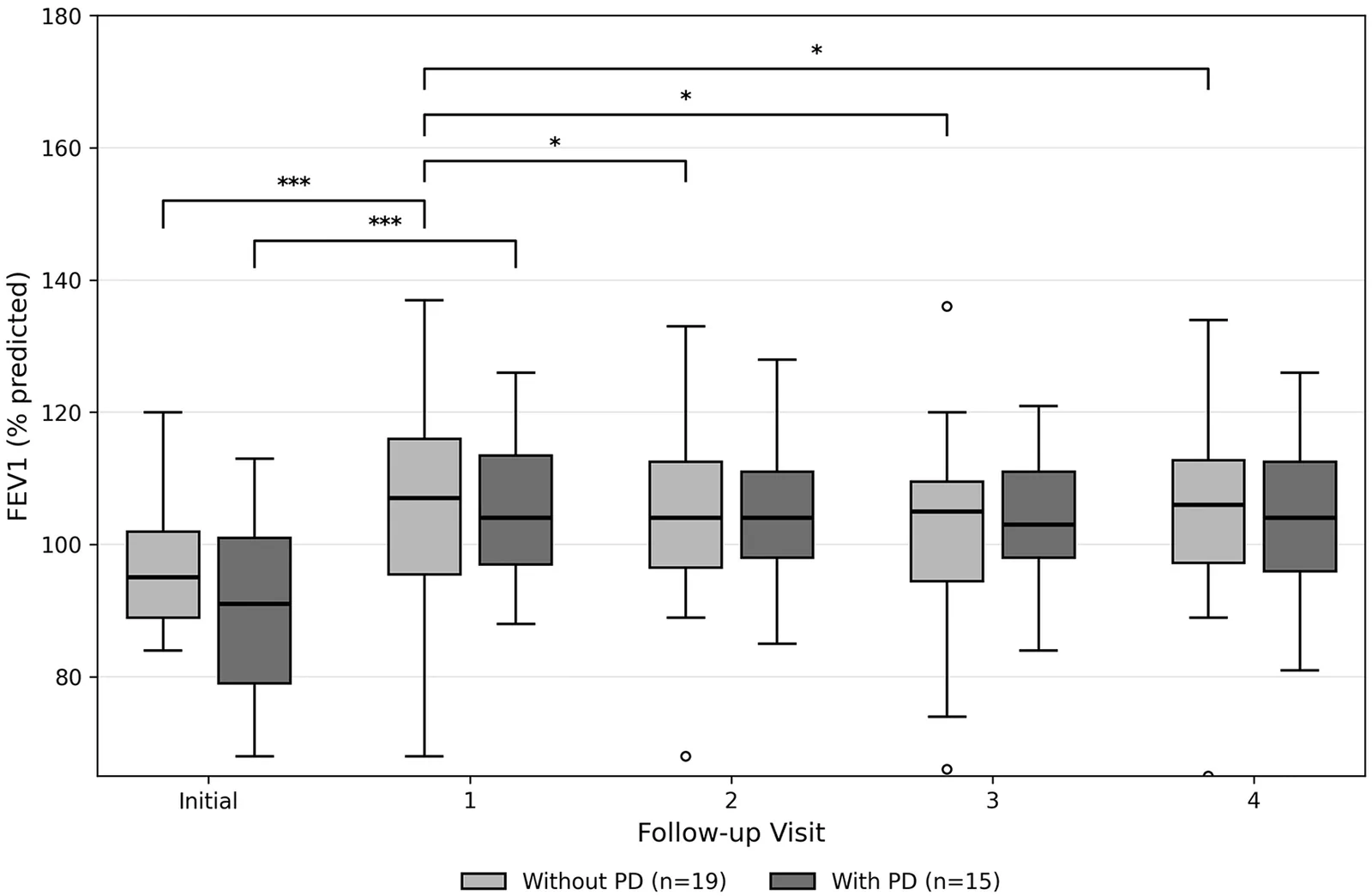
FEV1 as a percentage of the predicted value before and after starting ETI therapy DS, depressive symptoms(PHQ-9 score ≥7); *p < 0.05; ***p < 0.001.

Lung function parameters demonstrated sustained improvement following modulator initiation (Figure 3). Patients without depressive symptoms showed an increase in FEV1 (% predicted) from 95% (IQR 89–102) at baseline to 107% (IQR 96–116) at FV1 (*p* < 0.001), with subsequent small but statistically significant within-group decreases at FV2 (104%, IQR 96–112; *p* = 0.030), FV3 (105%, IQR 94–110; *p* = 0.014), and FV4 (106%, IQR 97–113; *p* = 0.041 vs. FV1) these pairwise comparisons were not corrected for multiplicity, and the 1%–3% differences fell within the test–retest variability of spirometry (exploratory, not clinically meaningful). In patients with depressive symptoms, FEV1 increased from 91% (IQR 79–101) at baseline to 104% (IQR 97–114) at FV1 (*p* < 0.001) and remained stable at subsequent visits (*p* = 0.278–0.806 vs. FV1). The Friedman test confirmed significant overall changes in both groups (*p* < 0.001).

### 3.4 Medical possession ratio

**Figure 4:**
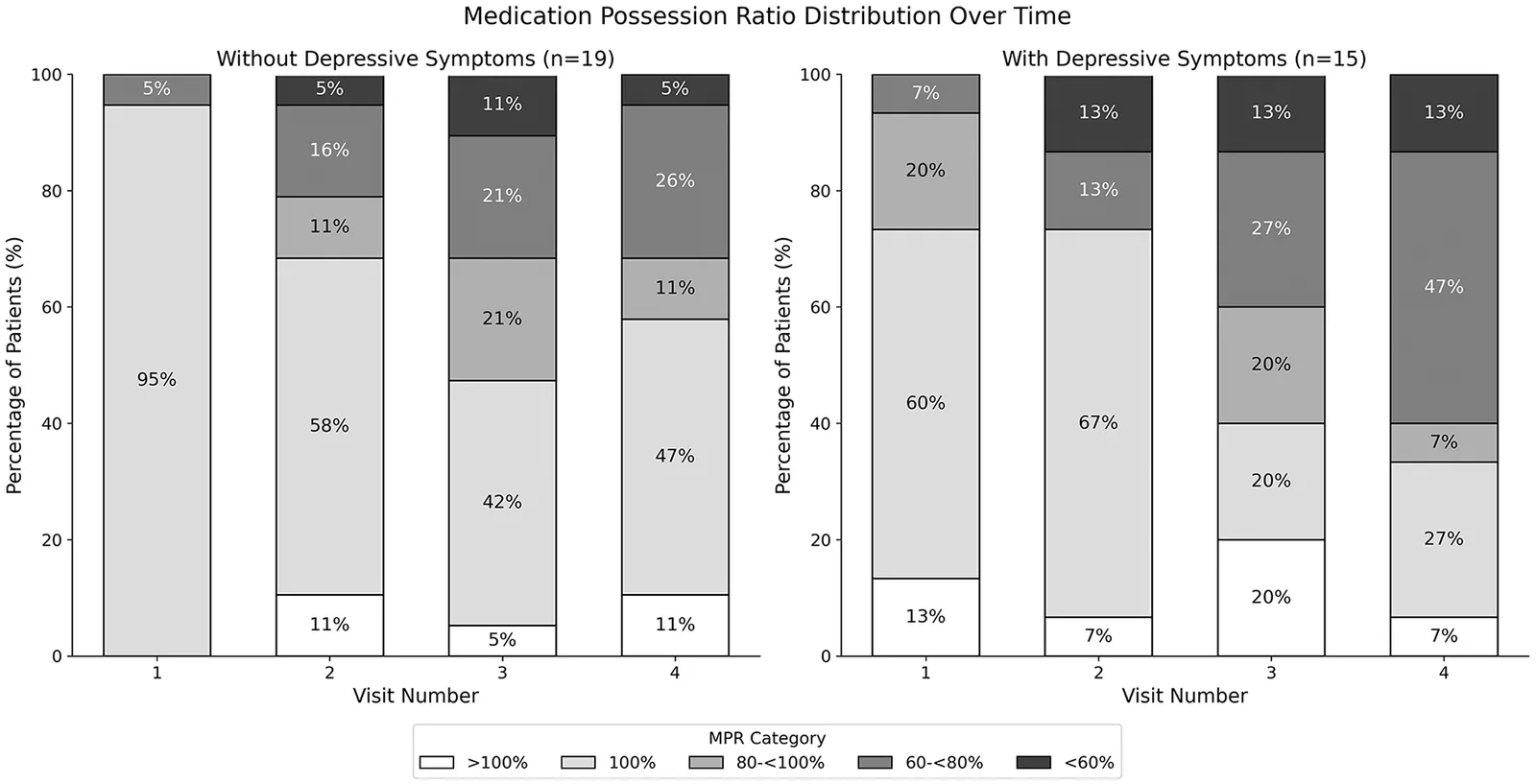
Follow-up medical possession ratio per visit and patient group.

Medication adherence, measured by the medication possession ratio (MPR), showed high initial values at FV1 in both groups (median 1.00, IQR 1.00-1.00 for patients without depressive symptoms; 1.00, IQR 0.92-1.00 for patients with psychological distress). A declining trend emerged over time in both cohorts (Figure 4). In patients without psychological distress, MPR decreased to 0.91 (IQR 0.71–1.00) at FV3 (*p* = 0.015 vs. FV1) and showed a trend toward further decline at FV4 (1.00, IQR 0.75–1.00; *p* = 0.058 vs. FV1). Similarly, patients with depressive symptoms demonstrated a trend toward decreased MPR at FV3 (0.83, IQR 0.66–1.00; *p* = 0.054 vs. FV1) and a statistically significant reduction at FV4 (0.75, IQR 0.66–1.00; *p* = 0.014 vs. FV1). The Friedman test revealed significant overall changes in both groups (without DS: *p* = 0.025; with DS: *p* = 0.044).

### 3.5 Pulmonary exacerbations

**Figure 5:**
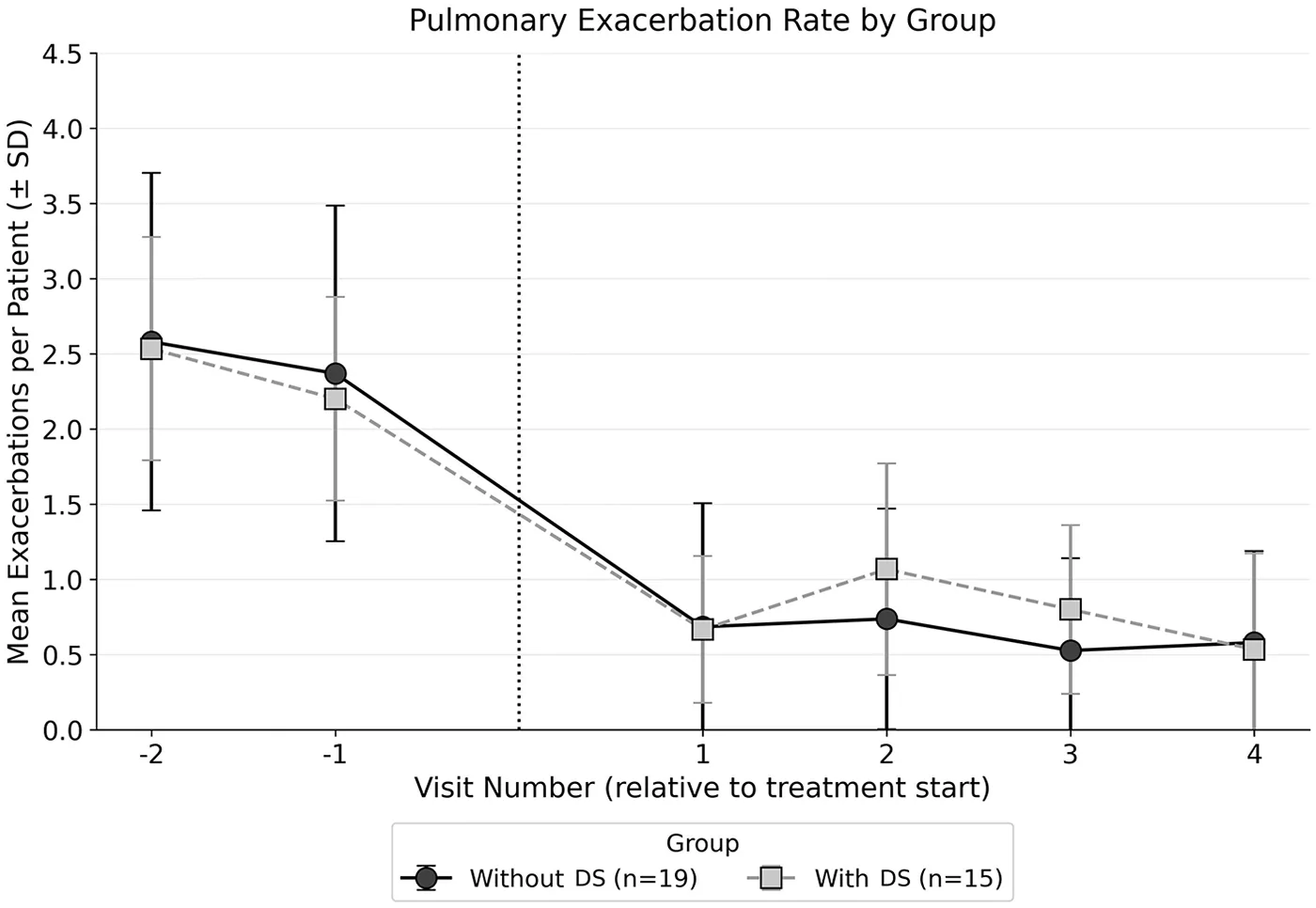
Pulmonary exacerbation rate per year DS, depressive symptoms (PHQ-9 score ≥7);.

The frequency of pulmonary exacerbations per period decreased substantially following modulator initiation (Figure 5). At baseline, both groups reported a median of 2.50 exacerbations per period (IQR 2.00–3.00 for patients without depressive symptoms; IQR 2.00–2.75 for patients with depressive symptoms). At FV1, exacerbation frequency decreased to 1.00 (IQR 0.00–1.00) in both groups (*p* < 0.001 vs. baseline). Throughout subsequent follow-up visits (FV1–4), exacerbation rates remained stable in patients without psychological distress (*p* = 0.180–0.782 vs. FV1), with FV3 showing the lowest median of 0.00 (IQR 0.00–1.00). In patients with depressive symptoms, exacerbations remained low but showed a trend toward increase at FV2 (1.00, IQR 1.00–1.50; *p* = 0.058 vs. FV1) before stabilizing at FV3 and FV4 (*p* = 0.317–0.480 vs. FV1). The Friedman test confirmed highly significant overall reductions in exacerbation frequency in both groups (*p* < 0.001).); because four longitudinal parameters were tested without correction for multiplicity, these omnibus results are considered exploratory.

### 3.6 Family burden

Familial burden factors were present in 15/34 participants (44.1%) and were numerically more frequent in the depressive-symptoms group (8/15, 53.3%) than in the group without (7/19, 36.8%; Fisher's exact *p* = 0.489; Table S3). Family burden alone was not significantly associated with SC, FEV₁ or MPR at any visit (all *p* > 0.05; Table S3). However, participants with both family burden and depressive symptoms (*n* = 8) had a significantly lower MPR at FV4 than all others (0.66 vs. 1.00; *p* = 0.038; Table S4). Given the small subgroup and multiple comparisons, this finding is exploratory and hypothesis-generating.

## 4 Discussion

This retrospective observational study examined the longitudinal relationship between depressive symptoms (DS) and adherence to elexacaftor/tezacaftor/ivacaftor (ETI) in 34 adolescents and young adults with cystic fibrosis (CF), with up to four years of follow-up. Three findings should be highlighted. First, ETI initiation led to rapid and sustained improvements in sweat chloride (SC) and FEV1 in both groups, consistent with the efficacy of ETI reported in pivotal trials and real-world cohorts (6, 7, 20). Second, 44.1% of patients exhibited DS (PHQ-9 ≥ 7). In this subgroup, SC values were more variable during long-term follow-up, with a marked increase at visit 4. Third, the medication possession ratio (MPR) in the depressive group declined significantly from 0.95 to 0.75 over the observation period, while FEV1 remained stable in both groups. Psychological comorbidity can therefore compromise both objective adherence indicators and pharmacodynamic treatment response, even under highly effective CFTR modulator therapy.

A methodological feature of this study is the use of SC not only as a measure of CFTR function but also as a complementary adherence indicator alongside pharmacy-derived MPR. SC is an established pharmacodynamic biomarker of CFTR activity, and the correlations between CFTR genotype, *in vitro* function, and clinical phenotype are well documented (21–23). In our cohort, SC decreased from approximately 101–105 mmol/L at baseline to 46–47 mmol/L at follow-up visit 1. This is comparable to the SC reduction reported in the phase 3 ETI trials (6, 7) and in real-world populations (20, 22). A new observation is that patients with depressive symptoms showed higher and more variable SC at follow-up visit 4 (within-group increase *p* = 0.012; between-group difference *p* = 0.056, a non-significant trend). Post-modulator SC heterogeneity has previously been attributed to genotype, drug exposure variability, drug-drug interactions, and the presence of complex alleles (21, 24). Our data are consistent with an additional contribution of depressive symptoms, plausibly mediated by adherence, although SC is a CFTR-function biomarker and this link remains an inference rather than a demonstrated mechanism. Rising SC values in a patient on stable ETI therapy should therefore prompt clinicians to consider not only pharmacokinetic or genetic explanations, but also psychological and behavioural factors, including non-adherence. Because the design is observational, a bidirectional relationship cannot be excluded: depressive symptoms may reduce adherence, and treatment burden or disease activity may, conversely, contribute to depressive symptoms.

The decline in MPR in the depressive subgroup is in line with existing evidence linking depression to lower adherence in CF. Several cross-sectional and longitudinal studies have shown that depressive symptoms are associated with reduced medication intake across CF treatment modalities, including inhaled therapies and pancreatic enzyme supplementation (10–12). Hilliard et al. (12) showed that medication beliefs mediate the relationship between depressive symptoms and adherence, pointing to a cognitive pathway by which psychological diseases can undermine treatment engagement. The challenge of maintaining adherence in adolescents with CF is not unique to the modulator era. In a five-year retrospective analysis of pre-modulator adherence based on pharmacy refill data, Shakkottai et al. (25) found that adolescents aged 13–21 years had consistently and significantly lower overall adherence than younger children. The lowest median refill rates were observed for pancreatic enzyme replacement therapy (46.2%) and CF multivitamins (58.3%). The youngest age group (0–5 years) maintained the highest adherence throughout the study period, which the authors attributed to greater parental supervision (25). Our data indicate that this age-related pattern of adherence decline persists under ETI therapy, and that psychological inbalance can exacerbate the developmental vulnerabilities of this transitional period (9, 26). The decline of MPR from 1.00 at Timepoint 1 to 0.75 at Timepoint 4 in patients with depressive symptoms is clinically relevant, since pharmacy refill-based MPR has been shown to correlate with subsequent health-care utilization and costs in CF (3).

FEV1 remained stable in both groups throughout follow-up, despite the declining MPR in the depressive subgroup. The non-depressive group showed a small but statistically significant within-group decrease in FEV1 at visits 2–4 relative to Timepoint 1 (107% → 104%–106% predicted; *p* = 0.014–0.041), which fell within spirometric test–retest variability and was not clinically meaningful. While FEV1 in depressed group remained statistically unchanged after the initial improvement (104% at Timepoint 1; *p* = 0.278–0.806 for subsequent comparisons). At first sight, this pattern appears paradoxical: the more adherent group shows a significant FEV1 decline, the less adherent group does not. The most plausible explanation is regression to the mean. The non-depressive group reached a supranormal level at Timepoint 1 (median 107% predicted), representing an overshoot at the time of maximal CFTR restitution and optimal early adherence. The subsequent modest decline to 104%–106% predicted likely reflects physiological settling towards a sustainable plateau rather than true deterioration, since values remained well above the pre-treatment baseline (95% predicted) throughout follow-up. The magnitude of this change (1–3 percentage points) falls within the test-retest variability of spirometry and is not clinically meaningful. The larger sample size of the non-depressed group (*n* = 19 vs. *n* = 15) may also have provided greater statistical power to detect these minor fluctuations, which could have been present but not statistically detectable in the smaller depressed subgroup.

Several additional factors may explain the overall stability of FEV1 despite declining adherence in the drepressed subgroup. First, FEV1 is a relatively insensitive measure of early or episodic lung function changes, in particular in patients with preserved lung function at baseline, as was the case in our cohort (median baseline FEV1 91%–95% predicted). More sensitive markers of ventilation inhomogeneity, such as the lung clearance index, might have revealed subclinical functional deterioration not captured by spirometry. Shakkottai et al. (25) likewise reported no significant association between adherence and lung function in their pre-modulator cohort, most likely because the majority of their participants had normal lung function throughout the observation period (mean FEV1 97% predicted). The insensitivity of spirometry to adherence-related effects therefore seems to be a consistent finding across the CF adherence literature in populations with preserved lung function. Second, the four-year observation window may have been too short to detect the cumulative downstream effects of intermittent non-adherence on lung function decline in a relatively young population with mild disease. Third, residual CFTR modulation under imperfect adherence may have been sufficient to maintain spirometric stability in the medium term, a hypothesis consistent with the potent pharmacodynamic effects of ETI even at suboptimal dosing. The significantly higher SC at FV 4 in the depressed group (a within-group increase; the between-group difference was a non-significant trend, *p* = 0.056), reflecting reduced CFTR function, may nevertheless indicate long-term pulmonary consequences that are not yet apparent in FEV1 trajectories. Extended follow-up is warranted.

The prevalence of depressive symptoms in our cohort (44.1%) is higher than the rates of moderate-to-severe depression (10%–26%) reported in PHQ-9 screening studies of adolescents with CF (27, 28). The use of a lower threshold (PHQ-9 ≥ 7) and a longitudinal maximum-score classification may partly account for this difference. Our findings are in line with the international TIDES study, which showed that depression and anxiety are elevated two- to threefold in the CF population compared with community samples (27). The Cystic Fibrosis Foundation and the European Cystic Fibrosis Society have issued consensus recommendations for annual mental health screening with the PHQ-9 and GAD-7 in all patients aged 12 years and older (8). Our data support the clinical relevance of these recommendations and indicate that even mild-to-moderate depressive symptoms, falling below conventional diagnostic thresholds for major depression, can have meaningful effects on adherence-related outcomes. Whether CFTR modulator therapy itself modifies the prevalence or trajectory of psychological inbalance remains under investigation. Some studies suggest stable or modestly improved mental health scores after ETI initiation (29, 30), while others have identified new or worsening neuropsychiatric symptoms in a subset of patients (26).

The temporal trajectory of adherence in our cohort deserves attention. Both groups showed near-perfect MPR in the first year of ETI therapy, followed by a gradual decline. This pattern is well described across chronic diseases and has recently been documented for CFTR modulators specifically. A systematic review by Hansen et al. (2) reported that adherence to CFTR modulators varies substantially across studies and tends to decline over time, with most investigators relying on pharmacy refill metrics similar to ours. Real-world data from Connett et al. (20) reported high adherence (proportion of days covered >90%) in adolescents over two years of ETI, but did not assess longer-term trends. The first three-year analysis of long-term adherence to ETI and the evening dose of ivacaftor (IVA), recently published by Hansen and colleagues (31), confirms and extends this pattern. In a cohort of 128 people with CF (mean age 28.8 years), aggregate adherence remained high across the observation period (median proportion of days covered 0.98 at year 1, 0.99 at year 2, and 0.98 at year 3 for ETI; comparable values for IVA). However, the proportion of non-adherent individuals (PDC <0.8) increased almost fourfold: from 3.3% to 12.5% for ETI and from 3.6% to 15.7% for IVA, with a statistically significant overall decline in PDC over time (*p* = 0.01 for ETI; *p* < 0.01 for IVA) (31). These results parallel the MPR trajectory in our psychologically depressed subgroup and show that even when group-level adherence appears high, a clinically meaningful minority of patients progressively disengages from therapy. The Hansen cohort was composed predominantly of adult patients (31), whereas our adolescent and young-adult cohort shows a comparable, and arguably steeper, decline in MPR confined to a psychologically vulnerable subgroup. This suggests that the temporal pattern of disengagement is both age- and psychosocially mediated. The decline of adherence over time is not unique to the modulator era. Shakkottai et al. (25) observed a decline in PERT adherence across all three age groups over a five-year period, with the 6–12 years group also showing a decrease in dornase alfa adherence from 80% to 67%. Sustained adherence is therefore difficult to maintain over time, regardless of the therapy, and the initial “honeymoon effect” of high adherence observed with novel therapies can attenuate progressively, in particular in psychologically vulnerable patients. Ongoing reinforcement of adherence-promoting strategies and structured monitoring are therefore essential to preserve the long-term benefits of ETI.

Another relevant aspect in the context of CF adherence is the relationship between nutritional status and treatment engagement. In our cohort, baseline characteristics were comparable between the two groups with respect to BMI, and the decline in adherence in the psychologically depressed group was not primarily driven by nutritional deterioration. The association between better nutritional status and improved adherence has, however, been documented in the pre-modulator literature. Shakkottai et al. (25) reported that a 10-percentile increase in BMI was significantly associated with greater odds of adherence across all medication categories except PERT (OR 1.11, 95% CI 1.07–1.14, *P* < 0.0001 for all medications combined). This positive association may reflect a broader pattern of health engagement: patients or families who are more proactive about nutrition may also be more adherent to medication regimens. In the ETI era, where weight gain is common, the interpretation of this association may shift. Future studies should examine whether ETI-associated nutritional improvements themselves reinforce or modify adherence behaviour.

A comparison with the five-year ivacaftor adherence study by Mitchell et al. (32) is informative. In 35 adults with the Gly551Asp mutation (mean age 29 years, baseline FEV1 58.4% predicted) receiving ivacaftor monotherapy, MPR declined by 2.5% per year (*p* = 0.007). This trajectory is consistent with the adherence attenuation observed in our ETI-treated adolescent cohort. The authors also showed a significant association between higher MPR and better-maintained FEV1 at 60 months (*p* = 0.036), providing direct evidence that adherence to CFTR modulators influences long-term pulmonary outcomes. Our study could not detect this association, most likely because of the considerably higher baseline lung function in our cohort (median FEV1 91%–95% vs. 58% predicted), which, as discussed above, limits the sensitivity of spirometry to adherence-related effects. The time-dependent decline in CFTR modulator adherence therefore appears to be consistent across different modulators, age groups, and disease-severity strata. This reinforces the need for sustained adherence monitoring and intervention strategies.

Several clinical implications follow from these findings. First, rising or fluctuating SC values during stable ETI therapy should alert clinicians to the possibility of non-adherence and trigger a comprehensive adherence assessment, including psychological screening. Second, routine annual PHQ-9 screening, as recommended by international guidelines (8), should be regarded not only as a mental health intervention but as an integral component of adherence monitoring in the modulator era. Third, integrated multidisciplinary care models that embed psychological support within the CF clinical team are particularly relevant for adolescents and young adults, who face specific developmental, psychosocial, and identity-related challenges (9, 26). The role of parental supervision in maintaining adherence among younger children (25) suggests that structured family-based interventions, including gradual and supported transfer of self-management responsibility, should be incorporated into transitional care programmes. Behavioural interventions informed by theory-based frameworks, such as the Theoretical Domains Framework (capability, opportunity, and motivation as determinants of adherence behaviour (4), offer a structured approach to intervention development. A Cochrane review noted, however, that the evidence for psychological adherence interventions in CF remains limited and heterogeneous, which highlights the need for well-designed trials in this area (1).

### 4.1 Limitations

Several limitations should be acknowledged. The retrospective, single-centre design and the relatively small sample size (*n* = 34) limit the generalizability of our findings and preclude definitive causal inference. The use of a single screening instrument (PHQ-9) for depressive symptoms does not capture the full spectrum of psychosocial burden, including anxiety, adjustment disorders, or externalizing behaviours, which may independently influence adherence. The dichotomization of depressive symptoms based on the maximum PHQ-9 score over follow-up, while pragmatic, does not capture the temporal dynamics of depression or its relationship with contemporaneous adherence fluctuations. Pharmacy refill–based MPR, although an objective and widely used adherence metric (2, 3), does not confirm actual medication ingestion and may overestimate true adherence—a limitation shared with all refill-based studies, including the five-year analysis by Shakkottai et al. (25), who noted that patients classified as non-adherent by specialty pharmacy data may in fact have obtained prescriptions from local pharmacies. SC variability may also reflect biological factors beyond adherence, including CYP3A4-mediated drug interactions, dietary fat intake affecting drug absorption, or intercurrent illness (21), 33. Classifying participants by the maximum PHQ-9 over follow-up uses later information to define an exposure applied to earlier outcomes, which precludes causal ordering and may overestimate the period prevalence of depressive symptoms. Multiple between- and within-subgroup comparisons were performed without correction for multiplicity, and the ≥10 and within-subgroup analyses were *post hoc*; these results are exploratory. SC differed between subgroups already at baseline, so part of the later divergence may reflect baseline imbalance and regression to the mean. The GAD-7 was not available, so anxiety could not be evaluated. A bidirectional relationship between depressive symptoms and adherence cannot be excluded.

Notwithstanding these limitations, our study is among the first to combine longitudinal MPR and SC data with standardized psychological screening in adolescents and young adults on ETI therapy over a four-year period.

Future research should employ prospective, multicentre designs with larger sample sizes to validate our findings and to delineate the causal pathways linking depressions, adherence, CFTR biomarker response, and long-term clinical outcomes. The integration of more sensitive outcome measures—including lung clearance index, chest imaging, and patient-reported outcome measures such as the CFQ-R—would provide a more comprehensive picture of the clinical consequences of adherence variability. Studies examining the longitudinal co-evolution of depressions and adherence using repeated measures at matched time points would help clarify whether depression is a cause, consequence, or both of declining adherence. Given the robust evidence from the pre-modulator era that adherence declines with age and with reduced parental involvement (25), interventions specifically targeting the adolescent transition period—incorporating digital health technologies, motivational interviewing, and collaborative care models—represent a critical translational priority.

## 5 Conclusion

In conclusion, this study demonstrates that depressive symptoms in adolescents and young adults with CF is associated with declining long-term ETI adherence as measured by MPR and greater variability in sweat chloride as a pharmacodynamic marker of CFTR function, despite preserved spirometric stability in the medium term. A substantial proportion of patients exhibited depressive symptoms, reinforcing the importance of systematic mental health screening in routine CF care. These findings are consistent with and extend the pre-modulator adherence literature, which has consistently identified adolescence as a period of heightened vulnerability for non-adherence (3, 25). The integration of SC monitoring, pharmacy refill data, and psychological assessment offers a multidimensional approach to adherence evaluation in the CFTR modulator era and may identify patients at risk of suboptimal treatment outcomes. Structured psychological support, targeted adherence interventions, and extended clinical follow-up are essential to ensure that the transformative potential of ETI therapy is fully realized for all patients with CF.

## Data Availability

The original contributions presented in the study are included in the article/Supplementary Material, further inquiries can be directed to the corresponding author.
