# Supplementary material for: Association of depressive symptoms with sweat chloride variability and medication possession ratio during long-term Elexacaftor/Tezacaftor/Ivacaftor therapy in adolescents and young adults with cystic fibrosis
Source: Front Pediatr. 2026 Aug 31;14:1888421. doi: 10.3389/fped.2026.1888421 (PMC13572717; doi:10.3389/fped.2026.1888421)
Supplement: Supplementary file 1 [file Table1.docx]

**Table S1 | Between-group comparison of sweat chloride, FEV₁, medication possession ratio and pulmonary exacerbations by PHQ-9 threshold (≥10 vs <10).**

| **Time point** | **PHQ-9 < 10 (n = 25)** | **PHQ-9 ≥ 10 (n = 9)** | **p** |
| --- | --- | --- | --- |
| **Sweat chloride (mmol/L) – median (IQR)** | | | |
| Baseline | 101.0 (95.0–103.0) | 107.0 (105.0–110.0) | **0.012** |
| FV1 | 42.0 (32.0–56.0) | 51.0 (36.0–63.0) | 0.482 |
| FV2 | 44.0 (35.0–55.0) | 44.0 (41.0–63.0) | 0.412 |
| FV3 | 54.0 (33.0–59.0) | 50.0 (40.0–69.0) | 0.769 |
| FV4 | 49.0 (39.0–64.0) | 56.0 (52.0–73.0) | 0.292 |
| **FEV₁ (% predicted) – median (IQR)** | | | |
| Baseline | 95 (89–102) | 91 (77–102) | 0.814 |
| FV1 | 107 (95–117) | 104 (97–110) | 0.953 |
| FV2 | 103 (97–113) | 109 (98–110) | 0.938 |
| FV3 | 104 (95–109) | 109 (101–115) | 0.249 |
| FV4 | 105 (97–113) | 104 (95–112) | 0.969 |
| **Medication possession ratio – median (IQR)** | | | |
| FV1 | 1.00 (1.00–1.00) | 1.00 (0.83–1.00) | **0.006** |
| FV2 | 1.00 (0.83–1.00) | 1.00 (1.00–1.00) | 0.704 |
| FV3 | 0.91 (0.66–1.00) | 0.83 (0.66–1.00) | 0.424 |
| FV4 | 1.00 (0.66–1.00) | 0.75 (0.66–1.00) | 0.686 |
| **Pulmonary exacerbations (per year) – median (IQR)** | | | |
| Baseline | 2.50 (2.00–3.00) | 2.00 (2.00–2.50) | 0.535 |
| FV1 | 1.00 (0.00–1.00) | 1.00 (0.00–1.00) | 0.659 |
| FV2 | 1.00 (0.00–1.00) | 1.00 (0.00–1.00) | 0.642 |
| FV3 | 1.00 (0.00–1.00) | 1.00 (1.00–1.00) | 0.158 |
| FV4 | 0.00 (0.00–1.00) | 1.00 (0.00–1.00) | 0.598 |

*Data are median (interquartile range). Subgroups were defined by the maximum PHQ-9 score over follow-up (<10 vs ≥10) and compared with the two-sided Mann–Whitney U test. Text in Bold = significant. Between-group differences reached significance only for baseline sweat chloride (p=0.012) and FV1 medication possession ratio (p=0.006); the FV4 sweat-chloride difference observed at the ≥7 threshold was not significant at ≥10 (p=0.292). Given the small ≥10 subgroup (n=9) and multiple comparisons, these sensitivity analyses are exploratory. FV, follow-up visit; FEV₁, forced expiratory volume in 1 s; IQR, interquartile range.*
