# Supplementary material for: Association of depressive symptoms with sweat chloride variability and medication possession ratio during long-term Elexacaftor/Tezacaftor/Ivacaftor therapy in adolescents and young adults with cystic fibrosis
Source: Front Pediatr. 2026 Aug 31;14:1888421. doi: 10.3389/fped.2026.1888421 (PMC13572717; doi:10.3389/fped.2026.1888421)
Supplement: Supplementary file 2 [file Table2.docx]

**Table S2 | Between-group comparison of sweat chloride, FEV₁ and medication possession ratio at each visit.**

| **Time point** | **Without DS (n = 19)** | **With DS (n = 15)** | **p** |
| --- | --- | --- | --- |
| **Sweat chloride (mmol/L) – median (IQR)** | | | |
| Baseline | 100.0 (95.5–104.0) | 105.0 (101.0–109.0) | 0.065 |
| FV1 | 46.0 (36.0–56.5) | 47.0 (31.0–60.5) | 0.755 |
| FV2 | 44.0 (35.5–52.0) | 44.0 (37.0–64.5) | 0.664 |
| FV3 | 51.0 (33.0–59.0) | 52.0 (43.0–61.5) | 0.627 |
| FV4 | 47.0 (37.5–58.5) | 56.0 (48.5–86.0) | 0.056 |
| **FEV₁ (% predicted) – median (IQR)** | | | |
| Baseline | 95 (89–102) | 91 (79–101) | 0.677 |
| FV1 | 107 (95.5–116) | 104 (97–113.5) | 0.862 |
| FV2 | 104 (96.5–112.5) | 104 (98–111) | 0.986 |
| FV3 | 105 (94.5–109.5) | 103 (98–111) | 0.768 |
| FV4 | 105 (95–112.5) | 104 (96–112.5) | 0.821 |
| **Medication possession ratio – median (IQR)** | | | |
| FV1 | 1.00 (1.00–1.00) | 1.00 (0.92–1.00) | 0.589 |
| FV2 | 1.00 (0.87–1.00) | 1.00 (0.83–1.00) | 0.905 |
| FV3 | 0.91 (0.71–1.00) | 0.83 (0.66–1.00) | 0.736 |
| FV4 | 1.00 (0.75–1.00) | 0.75 (0.66–1.00) | 0.111 |

*Data are median (interquartile range). Groups were compared with the two-sided Mann–Whitney U test. All between-group comparisons were non-significant (p > 0.05). DS, depressive symptoms (PHQ-9 ≥ 7); FV, follow-up visit; FEV₁, forced expiratory volume in 1 s; IQR, interquartile range.*
