# Supplementary material for: Association of depressive symptoms with sweat chloride variability and medication possession ratio during long-term Elexacaftor/Tezacaftor/Ivacaftor therapy in adolescents and young adults with cystic fibrosis
Source: Front Pediatr. 2026 Aug 31;14:1888421. doi: 10.3389/fped.2026.1888421 (PMC13572717; doi:10.3389/fped.2026.1888421)
Supplement: Supplementary file 3 [file Table3.docx]

**Table S3 | Sweat chloride, FEV₁ and medication possession ratio by family-burden status.**

| **Time point** | **Without family burden (n = 19)** | **With family burden (n = 15)** | **p** |
| --- | --- | --- | --- |
| **Sweat chloride (mmol/L) – median (IQR)** | | | |
| Baseline | 101 (97–105) | 103 (99–109) | 0.330 |
| FV1 | 46 (33.5–57) | 47 (33–60) | 0.958 |
| FV2 | 44 (33–54) | 44 (37–61.5) | 0.603 |
| FV3 | 46 (32–57) | 55 (48–64) | 0.092 |
| FV4 | 52 (35.5–60.5) | 54 (46.5–70) | 0.358 |
| **FEV₁ (% predicted) – median (IQR)** | | | |
| Baseline | 95 (88.5–102.5) | 95 (76–99.5) | 0.252 |
| FV1 | 107 (97.5–118.5) | 104 (96–109) | 0.340 |
| FV2 | 104 (99–114) | 101 (96.5–111.5) | 0.349 |
| FV3 | 107 (100–109.5) | 101 (93.5–111.5) | 0.455 |
| FV4 | 105 (97–112.5) | 104 (95–112.5) | 0.728 |
| **Medication possession ratio – median (IQR)** | | | |
| FV1 | 1.00 (1.00–1.00) | 1.00 (1.00–1.00) | 0.364 |
| FV2 | 1.00 (0.96–1.00) | 1.00 (0.74–1.00) | 0.889 |
| FV3 | 0.91 (0.71–1.00) | 0.91 (0.66–1.00) | 0.632 |
| FV4 | 1.00 (0.75–1.00) | 0.75 (0.66–1.00) | 0.203 |

*Data are median (interquartile range). Groups were compared with the two-sided Mann–Whitney U test. No comparison reached statistical significance (all p > 0.05; lowest p = 0.092 for sweat chloride at FV3). Family burden denotes the presence of at least one predefined familial burden factor (single-parent or separated household, socioeconomic strain, or parental mental health problems). FV, follow-up visit; FEV₁, forced expiratory volume in 1 s; IQR, interquartile range.*
