# Supplementary material for: Association of depressive symptoms with sweat chloride variability and medication possession ratio during long-term Elexacaftor/Tezacaftor/Ivacaftor therapy in adolescents and young adults with cystic fibrosis
Source: Front Pediatr. 2026 Aug 31;14:1888421. doi: 10.3389/fped.2026.1888421 (PMC13572717; doi:10.3389/fped.2026.1888421)
Supplement: Supplementary file 4 [file Table4.docx]

**Table S4 | Outcomes in patients with combined family burden and depressive symptoms versus all others.**

| **Time point** | **All others (n = 26)** | **Family burden + depressive symptoms (n = 8)** | **p** |
| --- | --- | --- | --- |
| **Sweat chloride (mmol/L) – median (IQR)** | | | |
| Baseline | 101.5 (98–105) | 104 (98.2–110.5) | 0.476 |
| FV1 | 48 (36–57.8) | 39.5 (29.5–54) | 0.350 |
| FV2 | 45 (36.5–54.5) | 39 (34–78.8) | 0.984 |
| FV3 | 51.5 (34.8–59) | 53.5 (44.5–65.2) | 0.583 |
| FV4 | 52 (40.2–63) | 60.5 (48–77.2) | 0.291 |
| **FEV₁ (% predicted) – median (IQR)** | | | |
| Baseline | 95 (89–102) | 87.5 (74.2–99.2) | 0.230 |
| FV1 | 106 (96.2–117) | 105.5 (97–108.5) | 0.626 |
| FV2 | 104 (97.2–112.8) | 105 (96–110.5) | 0.640 |
| FV3 | 104.5 (97.5–109.8) | 105 (96–112) | 0.887 |
| FV4 | 105.5 (97–112.8) | 102.5 (95–112.2) | 0.776 |
| **Medication possession ratio – median (IQR)** | | | |
| FV1 | 1.00 (1.00–1.00) | 1.00 (0.96–1.04) | 0.646 |
| FV2 | 1.00 (0.93–1.00) | 1.00 (0.62–1.00) | 0.245 |
| FV3 | 0.96 (0.68–1.00) | 0.74 (0.58–0.93) | 0.198 |
| FV4 | 1.00 (0.75–1.00) | 0.66 (0.65–0.81) | **0.038** |

*Data are median (interquartile range). Text in Bold = significant. Groups were compared with the two-sided Mann–Whitney U test. Patients with both family burden and depressive symptoms (PHQ-9 ≥ 7) showed a significantly lower medication possession ratio at FV4 (0.66 vs 1.00; asymptotic p = 0.038, exact p = 0.047); all other comparisons were non-significant. Given the small subgroup (n = 8) and multiple comparisons, this finding is exploratory and hypothesis-generating. Family burden denotes the presence of at least one predefined familial burden factor (single-parent or separated household, socioeconomic strain, or parental mental health problems). FV, follow-up visit; FEV₁, forced expiratory volume in 1 s; IQR, interquartile range.*
